# Stopover departure decisions in songbirds: do long-distance migrants depart earlier and more independently of weather conditions than medium-distance migrants?

**DOI:** 10.1186/s40462-020-0193-1

**Published:** 2020-02-07

**Authors:** Florian Packmor, Thomas Klinner, Bradley K. Woodworth, Cas Eikenaar, Heiko Schmaljohann

**Affiliations:** 1 Institute of Avian Research “Vogelwarte Helgoland”, An der Vogelwarte 21, 26386 Wilhelmshaven, Germany; 2 School of Natural Sciences, Bangor University, Deiniol Road, Bangor, LL57 2UW UK; 3 School of Biological Sciences, The University of Queensland, Brisbane, Queensland 4072 Australia; 4 Institute for Biology und Environmental Sciences, Carl von Ossietzky University of Oldenburg, Carl-von-Ossietzky-Straße, 26129 Oldenburg, Germany

**Keywords:** Departure decision, Departure timing, Fuel load, Long-distance migration, Medium-distance migration, Songbird, Weather conditions

## Abstract

**Background:**

Songbirds following distinct migration strategies (e.g. long- vs. short- to medium-distance migrants) often differ in their speed of migration during autumn and, thus, are assumed to face different time constraints. During migration, most songbird species alternate migratory flights with stopover periods. Many of them restrict these migratory flights to the night, i.e., they are nocturnal migrants. At stopover, nocturnal migrants need to select a specific night (night-to-night decision) and time of night (within-night decision) to resume migration. These departure decisions, which largely determine the speed of migration, are jointly affected by a set of intrinsic and extrinsic factors, i.e., departure cues. Here we aim to assess whether the set of intrinsic and extrinsic factors and the magnitude of their respective effects on stopover departure decisions differs between nocturnally migrating songbird species, depending on their migration strategy and associated time constraints.

**Methods:**

We radio-tracked migrating Northern Wheatears (*Oenanthe oenanthe*; long-distance migrant), European robins (*Erithacus rubecula*) and Common Blackbirds (*Turdus merula*; both medium-distance migrants) during autumn stopover and analysed their night-to-night and within-night departure timing in relation to intrinsic and extrinsic factors.

**Results:**

Species generally differed in their departure timing on both temporal scales, with shortest stopovers and earliest nocturnal departures in the long-distance migrant. Some factors, such as day of year, fuel load, cloud cover and crosswind, had consistent effects on stopover departure decisions in all three species. However, species differed in the effects of tailwind assistance, change in atmospheric pressure and air temperature on their stopover departure decisions. Whereas night-to-night decisions were affected by these extrinsic factors in either both or one of the medium-distance migrants, such effects were not found in the long-distance migrant.

**Conclusions:**

Our results suggest that the general timing of departures in songbirds is affected by the species-specific migration strategy and associated time constraints. Further, they imply that the assessment and usage of specific extrinsic factors, i.e., weather conditions, as departure cues is adjusted based on this migration strategy, with the long-distance migrants being least selective at departure. Other intrinsic and extrinsic factors, however, seem to be used as departure cues independent of migration strategy.

## Background

Billions of songbirds migrate between their breeding areas and wintering grounds by alternating periods of migratory flight with stopover periods that serve to rest and fuel. Since a songbird’s rate of energy accumulation is far slower than their rate of energy expenditure during flight [1, 2], they actually spend more time and energy during stopovers than during migratory flights [3, 4]. Total stopover duration, thus, strongly influences songbirds’ overall speed of migration [5, 6]. Travel speed, defined as total migration distance divided by total flight duration, is the other main determinant of overall speed of migration [7]. Many migratory songbirds restrict their migratory flights to the night (e.g. [8, 9]). Hence, their nocturnal departure timing defines the potential flight duration and the associated distance per flight bout [10, 11]. A bird’s decisions on which night to depart (night-to-night departure decision) and the exact time of departure within the night (within-night departure decision) thus directly affect its overall speed of migration and, consequently, its time of arrival at the migratory destination. Studying how individuals adjust these decisions advances our understanding of variation in arrival timing and its potential consequences for individual survival and fitness [12–14].

It is assumed that the fundamental spatiotemporal organisation of migration in songbirds is governed by an innate migration program, which provides the basis for timing, duration and direction of migration as well as for physiological adaptations associated with migratory behaviour (e.g. [15–18]). This innate migration program further defines the birds’ behavioural reaction norms to variation in different intrinsic and extrinsic factors (e.g. [19, 20]). There is substantial evidence that day of year (time within season; intrinsic factor), fuel load (energy stores; intrinsic factor), and prevailing weather conditions (extrinsic factors) affect night-to-night and within-night departure decisions and, thus, probably act as departure cues for migratory birds (e.g. [20–28]). The exact set of cues used by the birds and the magnitude of their respective effects seem to vary within- and between-species, probably because departure decisions are flexibly adjusted over time and space [19] and because species’ migration strategies differ in terms of time and/or energy constraints [29].

Migration is a complex phenomenon with remarkable inter- and intra-specific variation in the respective strategies. These strategies include both facultative and obligate migrations ranging over tens, hundreds or thousands of kilometres [30]. In the Africa-Eurasia flyway system songbirds performing obligate migrations are commonly categorised as either short- to medium-distance migrants (migrating predominantly within Eurasia), or long-distance migrants (migrating between Eurasia and tropical sub-Saharan Africa, i.e., trans-Saharan migrants). Long-distance migrants are assumed to face relatively strong time constraints to complete their migratory journey. Thus, they are probably selected to maximise speed of migration at the expense of relatively high energetic costs to reach their distant destinations in a reasonable time [1]. In short- to medium-distance migrants, by contrast, selection for the maximisation of speed is probably less pronounced and these birds may be more prone to minimize energy expenditure during migration, at least in autumn [29, 31, 32]. Accordingly, short- to medium-distance migrants and long-distance migrants should feature different behavioural reaction norms to intrinsic and extrinsic factors in autumn, i.e., they should differ in the use of specific cues for their departure decisions during stopover. There is some evidence that short- to medium-distance migrants travel, for example, with more favourable winds [29], but show a lower speed of migration than long-distance migrants [1, 33, 34]. So far, however, we lack detailed knowledge about how intrinsic and extrinsic factors may differently affect night-to-night and within-night departure decisions in birds facing different migration distances.

**Fig. 1:**
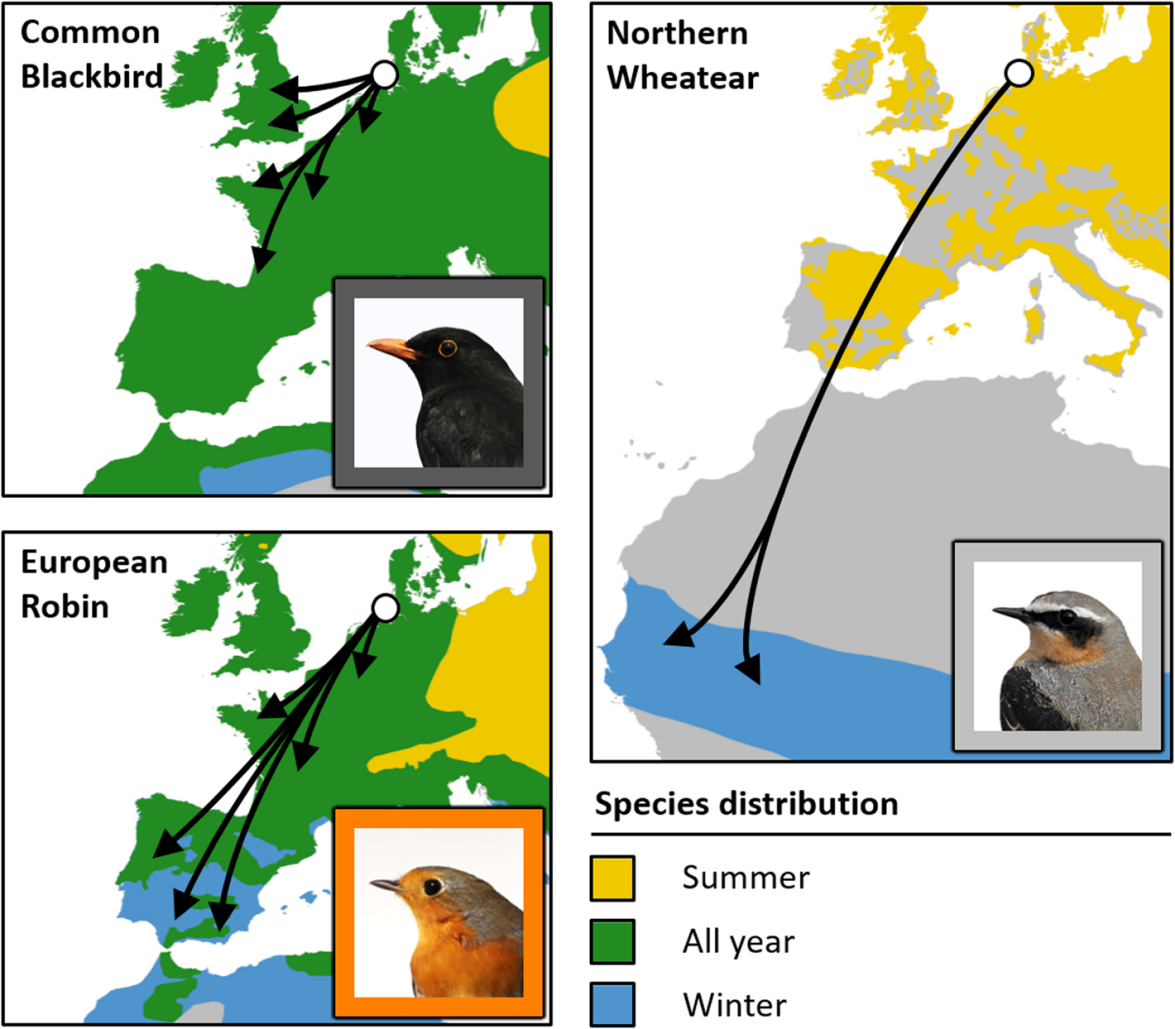
Hypothetical onward migration routes of Common Blackbirds, European Robins (both medium-distance migrants) and Northern Wheatears (long-distance migrants) departing from Helgoland (white dot) during autumn. Arrows figure simplified hypothetical migration routes towards the species-specific wintering grounds as derived from ring recoveries of birds ringed on Helgoland (Common Blackbirds and European Robins [35]) or estimated wintering locations from light-level geolocation data of European breeding birds (Norther Wheatear [36]). Maps represent an orthographic projection with Helgoland as the projection centre. Species distribution data were provided by BirdLife International [37]

Here, we investigate the timing of migratory departures in one long-distance migrant, the Northern Wheatear (*Oenanthe oenanthe*; Wheatear hereafter), and two medium-distance migrants, the European Robin (*Erithacus rubecula*; Robin hereafter) and the Common Blackbird (*Turdus merula*; Blackbird hereafter), from a coastal stopover site in Central Europe during autumn, when these species differ in distance to their migratory destinations (Fig. 1). All analyses are based on individual migratory departure data obtained by means of automated radio-telemetry (Motus Wildlife Tracking System [38]).

We expected that these species would differ in their general departure behaviour according to their migration strategy and associated time constraints. Hence, the long-distance migrant was expected to generally show shorter stopovers and, thus, a higher probability of leaving the stopover site each night (departure probability) as well as generally earlier departures within the night as compared to the medium-distance migrants. Further, we expected birds migrating late in the season, carrying large fuel loads and experiencing weather conditions favourable for migratory flights (e.g. tailwinds, clear skies, high atmospheric pressure), or unfavourable for an extended stopover (e.g. low air temperature) to show higher departure probability and earlier departures within the night than those migrating early in the season, carrying small fuel loads and experiencing opposing weather conditions. The particular effects of different weather variables on birds’ departure decisions were expected to be similar in direction among the species, as the decision-making processes should follow the same general principles independent of the migration strategy. Due to differences in the time constraints associated with the respective migration strategy, however, we expected these effects to differ in magnitude. Hence, departure decisions of the long-distance migrants should be less affected by weather conditions than those of the medium-distance migrants, as an increased selectivity for favourable weather probably prolongs the time spent at stopover and, thus, reduces the speed of migration.

## Methods

### Study site and study species

This study was conducted on Helgoland (54°11′N, 07°53′E), a small island in the North Sea, approx. 50 km off the German coastline. Many birds stop on Helgoland when crossing the North Sea during migration and use the island for resting and fuelling [35, 39]. We used spring traps baited with mealworms (*Tenebrio molitor*) to catch migrating Wheatears (*n* = 97) during August to October in 2015 and 2016. Migrating Robins (*n* = 54) and Blackbirds (*n* = 71) were caught with Helgoland traps during September to October 2017 and October to November 2016, respectively. Differences in catching methods and periods are justified by the species-specific differences in habitat use and autumn phenology on Helgoland [35]. Due to the very small size of the island (ca. 1 km^2^), we assume that the vast majority of birds was caught early during stopover (probably on the day of their arrival), independent of the catching method. Following capture, birds were aged based on plumage characteristics and/or the colouration of the upper mandible depending on the species [40]. We measured wing length (maximum chord [40];) to the nearest 0.5 mm and body mass to the nearest 0.1 g.

### Radio tracking

Birds were fitted with uniquely coded radio-transmitters (NTQB-1 Avian Nano Tag; weight: 0.29 g; burst interval: 2–4 s; Lotek Wireless Inc., Newmarket, ON, Canada) using leg-loop harnesses adjusted to body size [41]. Mass of radio-transmitters including harness (ca. 0.34 g) did not exceed 2% (Wheatears; min. Mass: 18.5 g), 2.6% (Robins; min. Mass: 14.4 g) or 0.5% (Blackbirds; min. Mass: 83.4 g) of the bird’s body mass, respectively. We used an automated digital radio-telemetry system consisting of four SensorGnome receivers (www.sensorgnome.org) located at three sites on Helgoland and equipped with a total of 12 radially aligned Yagi antennas ([20]; see Additional file 1). This radio-telemetry system continuously recorded radio signals during the study periods to determine the timing of individual departure events.

Departures as detected by this system are generally characterized by a rapid increase in signal strength detected from all/most antennas (bird is setting off the ground), followed by a decline in signal strength from a decreasing number of antennas until the loss of signal (bird is leaving the site towards a specific direction; see Additional file 1). All tracking data were inspected visually. If the specific departure pattern described above was missing, we did not ascertain departure timing. This was the case in 16 of the 97 Wheatears, 23 of the 54 Robins and 17 of the 71 Blackbirds radio-tagged for this study. Since we could not exclude that these birds were caught by a predator during stopover or that their radio-transmitters dropped or stopped transmitting (technical failure, battery life), they were omitted from all analyses. The bird’s nocturnal departure timing was defined as the time of highest signal strength during each departure event (see Additional file 1, Figure S1). Based on this timing we calculated the respective temporal difference between initial capture and departure (minimum stopover duration in days), the binary departure decisions of each bird for each day/night they stayed on Helgoland (staying vs. departing), as well as the bird’s nocturnal departure timing in relation to night length (proportion of night at departure). For the latter, a representation as proportion of night was necessary to compare the three species, which pass the study site during different periods in autumn and, thus, experience different night durations and sunset/sunrise times. In order to estimate the bird’s departure direction, we calculated a weighted circular mean of the directions the receiving antennas were aligned to (see Additional file 1 for details).

### Fuel load

In songbirds, energy stores used to fuel migratory flight primarily consist of accumulated fat and are commonly referred to as fuel loads. We assessed birds’ fuel loads at capture following the approach of Schmaljohann & Naef-Daenzer, 2011 [42]. Based on the individual body mass at capture and the estimated size-specific lean body mass we calculated each bird’s fuel load as follows:

1 $$ {fuel\ load}_{\mathrm{i}}=\frac{{body\ mass}_{\mathrm{i}}- lean\ {body\ mass}_{\mathrm{i}}}{lean\ {body\ mass}_{\mathrm{i}}} $$

We used wing length [mm] to estimate birds’ size-specific lean body mass [g], by means of species-specific linear regressions. Regarding Wheatears, we used results of the original regression by Schmaljohann & Naef-Daenzer, 2011 [42] that included 220 individuals with a fat score of 0–1 and a muscle score of 1 [41, 42] caught on Helgoland in the years 1998–2002 and 2008:

2 $$ \mathrm{Northern}\ \mathrm{Wheatear}: lean\ {body\ mass}_{\mathrm{i}}=0.29\  gm{m}^{-1}\ast {wing\ length}_{\mathrm{i}}-6.85\ g $$

(*n* = 220, F = 95.07, R^2^ = 0.30, *p* < 0.0001).

To estimate lean body mass of Robins and Blackbirds we considered “lean” individuals (fat score = 0, muscle score = 1–2 [43, 44];) caught on Helgoland between 2010 and 2017 yielding the following equations:

3 $$ \mathrm{European}\ \mathrm{Robin}: lean\ {body\ mass}_{\mathrm{i}}=0.21\  gm{m}^{-1}\ast {wing\ length}_{\mathrm{i}}-0.42\ g $$

(*n* = 151, F = 31.42, R^2^ = 0.17, *p* < 0.0001)

4 $$ \mathrm{Common}\ \mathrm{Blackbird}: lean\ {body\ mass}_{\mathrm{i}}=0.76\  gm{m}^{-1}\ast {wing\ length}_{\mathrm{i}}-12.08\ g $$

(*n* = 193, F = 28.53, R^2^ = 0.13, *p* < 0.0001)

### Weather data

We used NCEP reanalysis data provided by the National Oceanic and Atmospheric Administration (NOAA; Boulder, CO, USA; http://www.cdc.noaa.gov/cdc/data.ncep.reanalysis.derived.html; [45]) to estimate wind conditions individual birds experienced during their stopover on Helgoland, and at the time of their departure (see Additional file 1 for details). Based on these wind conditions we calculated the tailwind assistance [m/s] towards the species-specific mean departure direction (Wheatears: 176°; Robins: 138°; Blackbirds: 181°; see Additional file 1 for details) for each bird using the EQ^Tailwind^ [46] as follows:

5 $$ {tailwind\ assistance}_{\mathrm{i}}={windspeed}_{\mathrm{i}}\ast \cos \left( wind\  dir\mathrm{e}{ction}_{\mathrm{i}}- mean\ departure\ direction\right). $$

Additionally, we calculated the crosswind [absolute values in m/s] each bird experienced perpendicular to the species-specific mean departure direction (see above).

Other meteorological data were obtained from an automated weather station on Helgoland operated by the German Meteorological Office (DWD; ftp://ftp-cdc.dwd.de/pub/CDC/observations_germany/climate/hourly/). We used these measurements to assign atmospheric pressure [mbar], air temperature [°C], and cloud cover [x/8] at both time of sunset for each day a bird stayed on Helgoland and individual nocturnal departure time. As these data include hourly measurements, we assigned the last measurement before either sunset and/or departure. Further, we calculated change in atmospheric pressure and air temperature as the difference between the last measurement prior to either sunset or departure and the respective measurement 24 h before (see Additional file 1 for details on weather variables).

### Statistical analyses

All statistical analyses were conducted using the software R version 3.5.2 [47]. All continuous explanatory variables were scaled (z-transformed) on species level prior to modelling (for the original variation in these variables see Additional file 1: Table S1). We did not detect multicollinearity among the continuous explanatory variables (for all variance inflation factor < 1.76 [48]). In all modelling approaches with two or more explanatory variables included in the initial model, we conducted an automated model selection using the “dredge” function implemented in the R-package “MuMIn” [49]. Subsequent model averaging was performed for all models with a ΔAICc < 2 [50]. We used the “model.avg” function implemented in the R-package “MuMIn”. We provide average estimates and corresponding 95% confidence intervals for all explanatory variables included in the selected models with a ΔAICc < 2. If only one model met the selection criterion, we performed no averaging but provide estimates and 95% confidence intervals of this model. Visual inspection of standard diagnostic plots did not show any serious deviation from model assumptions in either of the models.

All modelling approaches described below were focused on assessing species-specific differences in the birds’ night-to-night and within-night departure decisions. In addition, we followed parallel modelling approaches focussing on strategy-specific differences in these departure decisions, with Wheatears representing the long-distance migration strategy, and Robins and Blackbirds together representing the medium-distance migration strategy (see Additional file 1).

#### Night-to-night departure decisions

We analysed whether the minimum stopover duration of Robins and Blackbirds differed from those of Wheatears using a Poisson regression model (generalised linear model) with species (categorical: three levels: Wheatear, Robin and Blackbird) as explanatory variable.

The effects of fuel load and weather variables on departure probability were analysed using two different modelling approaches. This was necessary, because all fuel load estimates were based on the birds’ body masses at capture and get less reliable with each day they spent at the study site. The modelling approach involving data on fuel load was, therefore, restricted to the departure probability during the first night following capture. Both modelling approaches are detailed below:

1. We assessed the effect of fuel load on departure probability during the first night following capture by fitting binary logistic regression models. The initial model included fuel load (continuous), species, day of year (1 January = 1; continuous), and the two-way interaction between fuel load and species as explanatory variables. Variables included in the selected models are detailed in Additional file 1: Table S2.
2. We assessed the effect of weather variables on night-to-night departure probability using time-dependent Cox proportional hazards models implemented in the “survival” package [51]. Cox proportional hazards models describe the probability of an event (here ‘departure’) occurring over time as a function of a baseline probability (hazard), which can be modified by a set of fixed or time-varying explanatory variables [52]. In contrast to others, these models provide the opportunity to include information about weather conditions birds experienced on nights they decided to depart and not to depart, which provides a thorough insight into factors shaping night-to-night departure decisions [26]. We estimated the departure probability as a function of species (fixed variable), day of year (time-varying variable), and a set of weather variables (time-varying variables). Weather variables included in the initial model were tailwind assistance (continuous), crosswind (continuous), cloud cover (proportional), atmospheric pressure (continuous), change in atmospheric pressure (Δ atmospheric pressure; continuous), air temperature (continuous), and change in air temperature (Δ air temperature; continuous). Additionally, the initial model included the two-way interactions between species and each of the different weather variables. Variables included in the selected models are detailed in Additional file 1: Table S3.

#### Within-night departure decisions

The effects of fuel load and weather variables on birds’ nocturnal departure timing were analysed in two different modelling approaches. The modelling approach involving data on fuel load was restricted to the nocturnal departure timing during the first night following capture for the same reason as described above. Both modelling approaches are detailed below:

1. We assessed the effect of fuel load on nocturnal departure timing (proportion of night at departure) of birds that left Helgoland during the first night following capture by fitting beta regression models using the “betareg” function implemented in the “betareg” package [53]. The initial model included fuel load, species, day of year, and the two-way interaction between fuel load and species as explanatory variables. Only the model including fuel load and species as explanatory variables met the criterion of our selection mentioned above.
2. We assessed the effects of weather variables on nocturnal departure timing (proportion of night at departure) by fitting beta regression models, which included birds leaving Helgoland during the first or any other night following capture. The initial model included species, day of year, tailwind assistance, crosswind, cloud cover, atmospheric pressure, Δ atmospheric pressure, air temperature, Δ air temperature, and the two-way interactions between species and each of the different weather variables. Variables included in the selected models are detailed in Additional file 1: Table S4.

## Results

### Night-to-night departure decisions

**Fig. 2:**
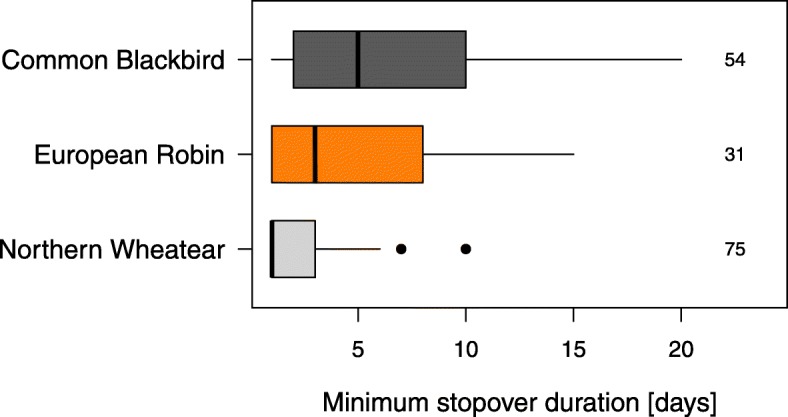
Variation in minimum stopover duration as observed in Northern Wheatears, European Robins and Common Blackbirds during autumn. Box plots show the 5th, 25th, 50th, 75th and 95th percentile as well as outliers (dots). Sample sizes are 75 (Northern Wheatear), 31 (European Robin) and 54 (Common Blackbird)

The three species differed significantly in their minimum stopover duration on Helgoland during autumn (Poisson regression model: Intercept: 0.75 (SE 0.08), *P <* 0.001; Robin: 0.81 (SE 0.11), *P* < 0.001; Blackbird: 1.07 (SE 0.1), *P* < 0.001; *n* = 160). Robins (median = 3 days; range = 1–15 days; *n* = 31) and Blackbirds (median = 5 days; range = 1–20 days; *n* = 54) were found to stay significantly longer than Wheatears (median = 1 day; range = 1–10 days; *n* = 75; Fig. 2).

**Fig. 3:**
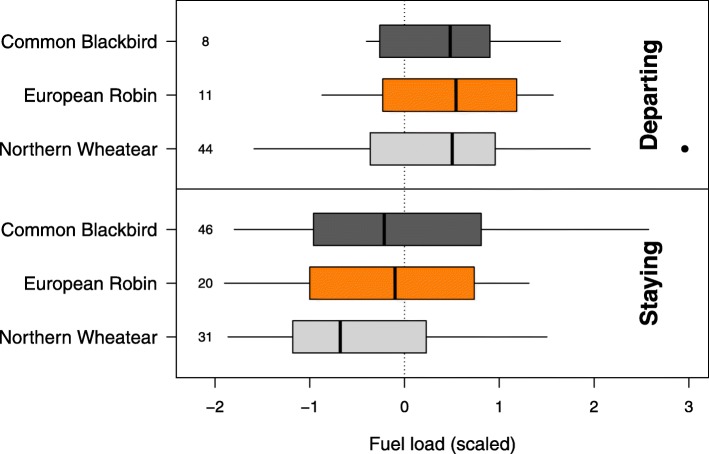
Fuel load of Northern Wheatears, European Robins and Common Blackbirds that departed the first night following capture (Departing) or that stayed longer at the stopover site (Staying). Box plots show the 5th, 25th, 50th, 75th and 95th percentile as well as outliers (dots). Numbers on the left side of boxes represent sample sizes. Please see Additional file 1: Table S1 for original variation in fuel load

Departure probability during the first night was positively correlated with fuel load in all species (Table 1, Fig. 3) and significantly higher in Wheatears (44 individuals, 58.7%) than in the two medium-distance migrants (Robins: 11 individuals, 35.5%; Blackbirds: 8 individuals, 14.8%; Table 1).

**Table 1:** Effects of fuel load and day of year on departure probability during the first night following capture in Northern Wheatears, European Robins and Common Blackbirds

Parameter	Estimate ± SE	95% CI	*p*
Intercept	0.395 ± 0.254	−0.108/0.897	0.124
Species (European Robin)	−1.142 ± 0.486	0.303/1.227	**0.020**
Species (Common Blackbird)	−2.399 ± 0.496	0.455/1.486	**< 0.001**
Fuel load	0.890 ± 0.215	−0.494/−0.072	**< 0.001**
Day of year	−0.257 ± 0.196	−0.643/0.130	0.193

Average model estimates, adjusted standard errors (SE), 95% confidence intervals (CIs) and associated *p*-values of parameters included in the candidate models in Additional file 1: Table S2 are shown. *P*-values < 0.05 are given in bold font. Reference category for species is Northern Wheatear

**Fig. 4:**
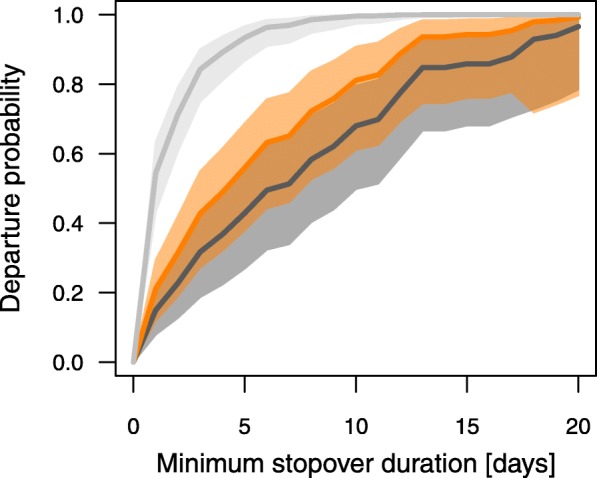
Night-to-night departure probability in Northern Wheatears (light grey), European Robins (orange) and Common Blackbirds (dark grey) predicted by a time-dependent Cox proportional hazards model. Species-specific predictions (lines) and associated 95% confidence intervals (shaded areas) are given. To illustrate the species-specific differences in the departure probability we used the candidate model with the best fit (Additional file 1: Table S3). Predictions were made with the remaining variables included in the model set to their mean

**Fig. 5:**
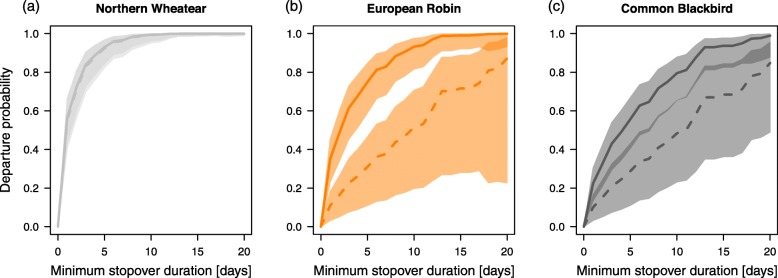
Night-to-night departure probability under different tailwind/headwind conditions in Northern Wheatears, European Robins and Common Blackbirds predicted by a time-dependent Cox proportional hazards model. Species-specific predictions (lines) and associated 95% confidence intervals (shaded areas) are given for the 25th percentile (broken line; light headwind) and 75th percentile (solid line; light tailwind) of the scaled tailwind assistance birds experienced at sunset. Details on the variation in tailwind assistance are given in Additional file 1: Table S1. To illustrate the effect of tailwind assistance on departure probability we used the candidate model with the best fit (Additional file 1: Table S3). Predictions were made with the remaining variables included in the model set to their mean

Considering the entire stopover period of the birds revealed a similar pattern, with Robins and Blackbirds showing a significantly lower night-to-night departure probability than Wheatears, confirming the results from the Poisson model (Table 2; Fig. 4). Day of year had a positive effect and cloud cover had a negative effect on the night-to-night departure probability in all species (Table 2). The significant two-way interaction between species and tailwind assistance indicated that the night-to-night departure probability of Robins and Blackbirds, but not Wheatears, was affected by tailwind assistance, with higher departure probability under tailwind than under headwind conditions (Table 2; Fig. 5). Crosswind had a negative effect on the night-to-night departure probability in all species (Table 2). The significant two-way interaction between species and Δ atmospheric pressure indicated that Robins, but not Wheatears and Blackbirds, had a lower departure probability when the atmospheric pressure increased compared to the day before (Table 2). The significant two-way interaction between species and air temperature indicated that departure probability of Blackbirds, but not Wheatears and Robins, was affected by air temperatures experienced during stopover. Blackbirds showed a higher departure probability under colder conditions (Table 2).

**Table 2:** Effects of weather variables and day of year on night-to-night departure probability in Northern Wheatears, European Robins and Common Blackbirds

Parameter	Estimate ± SE	95% CI	*p*
Species (European Robin)	−1.112 ± 0.240	−1.581/−0.642	**< 0.001**
Species (Common Blackbird)	−1.531 ± 0.243	−2.008/− 1.054	**< 0.001**
Cloud cover	−0.171 ± 0.078	−0.324/− 0.017	**0.029**
Tailwind assistance	0.100 ± 0.134	−0.173/0.351	0.506
Crosswind	−0.324 ± 0.105	−0.530/− 0.120	**0.002**
Atmospheric pressure	0.166 ± 0.100	−0.030/0.362	0.098
Δ atmospheric pressure	0.138 ± 0.126	−0.108/0.384	0.273
Air temperature	0.340 ± 0.173	0.000/0.680	0.050
Day of year	0.330 ± 0.108	0.118/0.542	**0.002**
Species (European Robin) x tailwind assistance	0.644 ± 0.266	0.123/1.165	**0.015**
Species (Common Blackbird) x tailwind assistance	0.550 ± 0.215	0.128/0.972	**0.011**
Species (European Robin) x Δ atmospheric pressure	−0.617 ± 0.269	−1.144/− 0.089	**0.022**
Species (Common Blackbird) x Δ atmospheric pressure	−0.013 ± 0.181	−0.369/0.343	0.943
Species (European Robin) x air temperature	−0.088 ± 0.293	−0.663/0.487	0.765
Species (Common Blackbird) x air temperature	− 0628 ± 0.216	−1.051/− 0.205	**0.004**

Average model estimates, adjusted standard errors (SE), 95% confidence intervals (CIs) and associated *p*-values of parameters included in the candidate models in Additional file 1: Table S3 are shown. *P*-values < 0.05 are given in bold font. Reference category for species is Northern Wheatear

### Within-night departure decisions

**Fig. 6:**
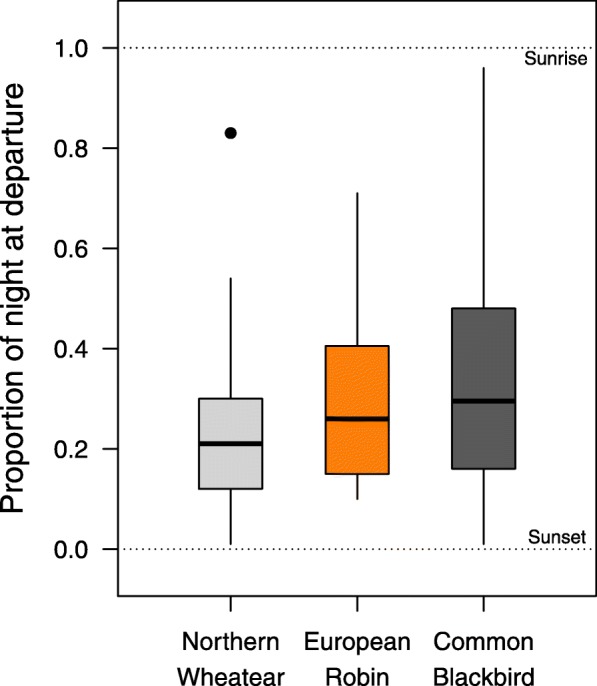
Variation in nocturnal departure timing as observed in Northern Wheatears, European Robins and Common Blackbirds during autumn. Nocturnal departure timing is expressed as proportion of night at departure. Box plots show the 5th, 25th, 50th, 75th and 95th percentile as well as outliers (dots). Sample sizes are 75 (Northern Wheatear), 31 (European Robin) and 54 (Common Blackbird)

**Fig. 7:**
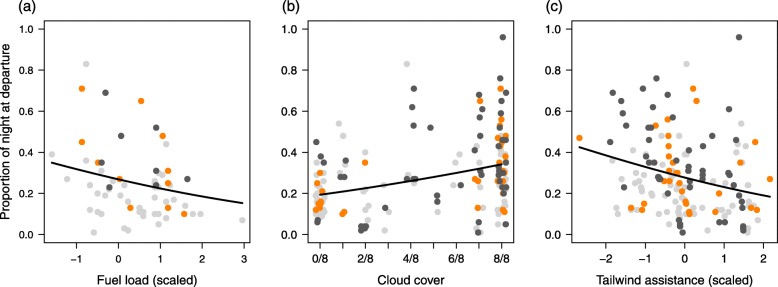
Effects of (**a**) fuel load, (**b**) cloud cover and (**c**) tailwind assistance on nocturnal departure timing (proportion of night at departure) obtained from two beta regression models (Tables 3 and 4). Shown are the respective model predictions (black lines) and the raw data (dots) for Northern Wheatears (light grey), European Robins (orange) and Common Blackbirds (dark grey). To illustrate the effects of fuel load, cloud cover and tailwind assistance we used the respective candidate models with the best fit (Additional file 1: Table S3). The remaining variables included in the respective model were set to their mean and species was not considered. Please see Additional file 1: Table S1 for original variation in fuel load and tailwind assistance

Nocturnal departure timing, measured as the proportion of night at departure, differed significantly between the species. Robins (median = 0.26; range = 0.10–0.71; *n* = 31) and Blackbirds (median = 0.30; range = 0.01–0.96; *n* = 54) were found to depart significantly later within the night than Wheatears (median = 0.21; range = 0.01–0.83; *n* = 75; Tables 3 and 4; Fig. 6 and Additional file 1: Figure S2). Variation in nocturnal departure timing differed significantly between Wheatears and Blackbirds (Fligner-Killeen Test: median χ^2^ = 11.62; df = 1; *P* < 0.001), with more variation in the Blackbirds, but not between Wheatears and Robins (Fligner-Killeen Test: median χ^2^ = 1.96; df = 1; *P* = 0.16). Fuel load had a significant negative effect on nocturnal departure timing during the first night, with relatively large fuel loads yielding early departures in all species (Table 3, Fig. 7a). Further, nocturnal departure timing was positively affected by cloud cover and negatively affected by tailwind assistance (Table 4, Fig. 7b and c). Birds that experienced clear skies and tailwinds departed in general earlier within the night than birds that experienced overcast skies and headwinds (Table 4, Fig. 7b and c).

**Table 3:** Effect of fuel load on nocturnal departure timing (proportion of night at departure) during the first night following capture in Northern Wheatears, European Robins and Common Blackbirds

Parameter	Estimate ± SE	95% CI	*p*
Intercept	−1.284 ± 0.124	− 1.527/− 1.040	**< 0.001**
Species (European Robin)	0.765 ± 0.236	0.303/1.227	**0.001**
Species (Common Blackbird)	0.971 ± 0.263	0.455/1.486	**< 0.001**
Fuel load	−0.283 ± 0.108	−0.494/− 0.072	**0.009**

Estimates, standard errors (SE), 95% confidence intervals (CIs) and associated *p*-values of all parameters included in the final model are shown. *P*-values < 0.05 are given in bold font. Reference category for species is Northern Wheatear

**Table 4:** Effects of weather variables and day of year on nocturnal departure timing (proportion of night at departure) in Northern Wheatears, European Robins and Common Blackbirds

Parameter	Estimate ± SE	95% CI	*p*
Intercept	−1.171 ± 0.097	−1.361/− 0.981	**< 0.001**
Species (European Robin)	0.356 ± 0.169	0.025/0.687	**0.035**
Species (Common Blackbird)	0.379 ± 0.142	0.100/0.657	**0.008**
Cloud cover	0.238 ± 0.084	0.072/0.403	**0.005**
Tailwind assistance	−0.213 ± 0.090	− 0.388/− 0.037	**0.020**
Crosswind	− 0.042 ± 0.065	− 0.170/0.090	0.517
Atmospheric pressure	−0.052 ± 0.072	−0.193/0.090	0.473
Δ air temperature	0.057 ± 0.068	−0.076/0.190	0.400
Day of year	−0.042 ± 0.067	−0.174/0.089	0.530
Species (European Robin) x cloud cover	0.218 ± 0.176	−0.127/0.564	0.216
Species (Common Blackbird) x cloud cover	0.211 ± 0.145	−0.073/0.496	0.146
Species (European Robin) x tailwind assistance	0.085 ± 0.171	−0.251/0.421	0.620
Species (Common Blackbird) x tailwind assistance	−0.259 ± 0.149	−0.551/0.032	0.082

Average model estimates, adjusted standard errors (SE), 95% confidence intervals (CIs) and associated *p*-values of parameters included in the candidate models in Additional file 1: Table S4 are shown. *P*-values < 0.05 are given in bold font. Reference category for species is Northern Wheatear

## Discussion

This study revealed longer stopovers in two medium-distance migrants (Robin and Blackbird) than in a long-distance migrant (Wheatear) during autumn migration (Fig. 2). Day of year, fuel load, cloud cover and crosswind had consistent effects on night-to-night departure decisions and the resulting departure probabilities in all three species (Tables 1 and 2, Fig. 3). Other parameters, like tailwind assistance, change in atmospheric pressure and air temperature, however, had effects on night-to-night departure decisions in both or one of the medium-distance migrants only (Table 2, Fig. 5). In contrast to night-to-night departure decisions, tailwind assistance affected within-night departure decisions and the resulting nocturnal departure timing in all three species in a similar way. Further, there was a consistent effect of fuel load and cloud cover on nocturnal departure timing (Tables 3 and 4). Nocturnal departure timing, however, generally differed between the long-distance migrant and the two medium-distance migrants, with the former setting off earlier within the night (Tables 3 and 4, Fig. 6). Based on these results we propose that group-specific differences in speed of migration between long- and medium-distance migrants [1, 33, 34] may be explained by group-specific differences in the night-to-night and within-night departure decision-making process. These differences, in turn, can partly be attributed to a differential selectivity for favourable weather conditions at departure between these two groups.

### General differences between species and effect of time within the season

Variation in total stopover duration and, to a lesser extent, variation in nocturnal departure timing, generally affect variation in the overall speed of migration [5–7, 10, 54]. Songbirds’ stopover departure decisions, thus, should conform to the time constraints associated with the respective species- or population-specific migration strategy. Here we show that stopover departure decisions generally differed between the three investigated songbird species in accordance with their different migration strategies. The behaviour of the long-distance migrant, which showed shortest stopovers, highest departure probabilities and performed earliest departures within the night (Figs. 2, 4 and 6), supports the time-minimising (or speed-maximising) strategy as proposed by optimal bird migration theory [1, 32]. The two medium-distance migrants, in contrast, showed relatively longer stopovers, lower departure probabilities and later departures within the night suggesting a generally slower pace during migration. Besides these differences, there was a consistent effect of day of year on night-to-night departure decisions, with higher departure probabilities and shorter stopovers towards the end of the respective migration season in all three species. This result is in line with previous studies (e.g. [21]). It can be generally assumed that birds migrating late in the season face increased time constraints as compared to their conspecifics migrating early. Migratory songbirds, thus, may generally adjust their strategy towards minimising the time spent at stopover towards the end of the season [55]. This would require behavioural adjustments at the respective stopover sites, which are likely to come with higher costs for energy and safety in the sense of optimal migration theory [1, 32].

### Effects of weather conditions (extrinsic factors)

Our results suggest that differences in night-to-night departure decisions between the one long- and two medium-distance migrant species are at least partly explained by a differential response to extrinsic factors like wind, change in atmospheric pressure and air temperature.

Wind is generally considered as a major determinant of birds’ migratory behaviour because it influences the current local flight conditions and associated energetic costs of transport and provides information about flight conditions to be expected in the near future [56, 57]. In this study, we found that night-to-night departure decisions of the medium-distance migrants, Robins and Blackbirds, were affected by tailwind assistance, with higher departure probabilities under tailwind than under headwind conditions. This was not the case in the Wheatears (Fig. 5), which again is in line with the time-minimising strategy suggested for long-distance migrants (e.g. [1]). Crosswind, in contrast, affected the night-to-night departure decisions of all three species alike and generally lowered birds’ departure probabilities. We propose that during stopover migrants may generally face a trade-off between waiting for favourable tailwind conditions to minimize their energetic costs of transport and resuming migration as soon as they are physiologically capable to minimize stopover duration and, thus, increase their overall speed of migration. The individual bird’s assessment of such a trade-off and the resulting night-to-night departure decision could be driven by its general migration strategy and the associated time constraints. The apparent avoidance of crosswind at departure, however, appears to be strategy-independent based on the current data. Such a general response to crosswind in night-to-night departure decisions would help birds to minimise lateral drift, which, if unintended, could be fatal during the sea crossing following a departure from Helgoland. Within-night departure decisions were found to be affected by tailwind assistance in all three species alike. This suggests that the observed effect of tailwind conditions on within-night departure decisions may represent a more general mechanism, which seems independent of the respective migration strategy. In fact, once a bird has decided to depart on a given night, it will benefit from relatively early departures under favourable wind conditions. Benefits include both an increase in the potential nocturnal flight duration and flight distance [10] and a decrease in the associated energetic costs of transport.

Changes in atmospheric pressure were found to affect night-to-night departure decisions in one of the two medium-distance migrant species. Robins were found to be less likely to depart when atmospheric pressure increased compared to the previous day. This is contrary to the results of previous studies reporting higher departure probabilities in migratory songbirds in response to increases in atmospheric pressure [26, 31, 58]. Birds are assumed to use atmospheric pressure and its changes as cues to predict favourable flight conditions for migration and respond accordingly, with high and/or increasing atmospheric pressure often promoting departure [59–61]. Beyond that, atmospheric pressure may alter the foraging conditions at stopover for insectivorous birds, because it is known to affect the activity patterns of insects [62]. Therefore, an effect of changes in atmospheric pressure on birds’ night-to-night departure decisions could potentially be based on associated changes in insect activity and, thus, food availability at stopover. In fact, it has been demonstrated that a bird’s behavioural response to atmospheric pressure can change over the course of its migratory journey [19]. This implies flexible adjustments in the assessment of this cue for the individual stopover departure decision.

Air temperatures also had an effect on night-to-night departure decisions in one of the two medium-distance migrant species. Blackbirds showed a higher departure probability when air temperatures near the surface were relatively low, which is in line with previous studies [4, 19, 23]. It is known that birds can reduce energetic costs associated with thermoregulation by avoiding low air temperatures during stopover [3]. Thus, low air temperatures should promote departure from stopover, especially during autumn migration when warmer conditions can be expected towards the southerly migratory destination. Blackbirds were probably more responsive to air temperatures than the other species, because they experienced, on average, lowest temperatures during stopover (Additional file 1: Table S1). Previous studies demonstrated a similar response of Wheatears to variation in air temperatures [4, 19, 23]. Probably, Wheatears and Robins considered in this study did not experience air temperatures low enough to promote the corresponding behavioural response, i.e., departure from stopover.

Cloud cover, as opposed to the other weather variables considered in this study, was found to have a consistent effect on both night-to-night and within-night departure decisions in all three species. Birds generally had a higher departure probability and departed earlier within the night under clear skies than under overcast conditions, which is in line with the results of other studies ([26, 28, 58, 63, 64] but see [61, 65]). Two mutually non-exclusive causes seem conceivable to explain this general pattern. First, migrants may generally postpone their departures if the conditions for nocturnal orientation are suboptimal, i.e., celestial cues (e.g. stars) required for orientation are not visible (e.g. [21, 26, 28, 58]). Second, overcast conditions are usually associated with an increased probability of precipitation, which in turn represents a hazard during flight [66]. Migrants, thus, should consider the probability of precipitation for their departure decision and respond accordingly. We argue that cloud cover seems to have a general effect on stopover departure decisions irrespective of the migration strategy, because of its consequences for birds’ orientation ability and survival probability.

### Effect of fuel load (intrinsic factor)

Fuel is required for locomotion and, thus, limits the potential duration and distance of a bird’s migratory flight (e.g. [27]). Therefore, the individual fuel load and its recent changes are considered fundamental cues for birds’ stopover departure decisions and the adjustments of migratory behaviour (e.g. [1, 10, 27, 67–69]). Furthermore, fuel loads acquired at stopover carry-over to influence the spatio-temporal progress of migration in songbirds across continents [70]. Our results are in line with most previous studies (reviewed in [10, 27]), with relatively large fuel loads promoting early departures during the first night following capture on both temporal scales, both in the one long-distance and the two medium-distance migrant species. This suggests that the adjustments of birds’ individual stopover departure decisions with regard to fuel load seem to follow a common principle independent of the species-specific migration strategy and the associated time constraints. Exceptions from this principle are scarce and usually coincide with specific geographical circumstances (e.g. songbirds encountering an ecological barrier, like the sea) potentially masking the direct effect of fuel (e.g. [20, 21]). Thus, we assume that migratory songbirds generally consider their fuel loads in stopover departure decisions on both temporal scales. Additionally, they integrate a number of other factors (see above) in the decision-making process, which may act as departure cues depending on the specific migration strategy and the respective geographical and ecological context.

## Conclusions

The results of this study are based on radio-telemetry data obtained from migrating songbirds caught at an island stopover site and tracked until their individual departure. Therefore, we have no information on the exact locations of the birds’ actual breeding areas and wintering grounds and, hence, their individual migration distances. Further, we lack information on the spatio-temporal progress during preceding stages of their journey. Instead, we are limited to allocating the birds to distinct groups based on the estimated average migration strategies of populations passing our study site (see Fig. 1). Despite these limitations, our results generally support the hypothesis that differences in migration strategies and associated time constraints affect stopover departure decisions of migratory songbirds. We found that in a long-distance migrant, the average stopover duration was shorter and departures occurred, on average, earlier within the night than in two medium-distance migrants. Stopover departure decisions were found to be affected by a set of intrinsic and extrinsic factors, both on the night-to-night and within-night scale. Some factors had consistent effects on stopover departure decisions in all three investigated species. Species, however, differed in the effect of certain extrinsic factors, i.e., weather conditions, on night-to-night departure decisions, with the long-distance migrant species generally being least affected. This may imply a differential selectivity for favourable weather at departure between long- and medium-distance migrants. Based on the current data, we suggest that migratory songbirds adjust their assessment of specific weather conditions as departure cues based on their migration strategy and the associated time constraints. Nonetheless, similar studies on other migrant species and at other stopover sites are required to test the generality of our results and conclusions. Finally, we stress the importance of interpreting data on songbirds’ nocturnal migratory behaviour, including stopover departure decisions, in the respective ecological and geographical context of the landscapes and habitats they traverse. Individual behavioural responses to certain intrinsic and extrinsic factors may change along the migration route and over the course of the season [19, 20].

## Supplementary information

**Additional file 1: **Additional methods: Radio tracking and weather data. **Figure S1.** Radio-telemetry system on Helgoland and example of signals received during the departure of a radio tracked northern wheatear. **Figure S2.** Variation in nocturnal departure timing as observed in Northern Wheatears, European Robins and Common Blackbirds during autumn. **Table S1.** Variation in explanatory variables used to explain night-to-night and within-night departure decisions in the three investigated migratory songbird species. **Table S2.** Comparison of candidate binary logistic regression models to assess the effect of fuel load on birds’ departure probability during the first night following capture (night-to-night departure decision). **Table S3.** Comparison of candidate time-dependent Cox proportional hazards models to assess the effects of weather variables on birds’ departure probability (night-to-night departure decision). **Table S4.** Comparison of candidate beta regression models to assess the effects of weather variables on birds’ nocturnal departure timing (proportion of night at departure; within-night departure decision). Strategy-specific differences in birds’ night-to-night and within-night departure decisions: Modelling approaches, Results.

## Data Availability

We intend to provide all relevant data in the Dryad Digital Repository.
