# Supplementary material for: Stopover departure decisions in songbirds: do long-distance migrants depart earlier and more independently of weather conditions than medium-distance migrants?
Source: Mov Ecol. 2020 Feb 7;8:6. doi: 10.1186/s40462-020-0193-1 (PMC7006082; doi:10.1186/s40462-020-0193-1)
Supplement: Supplementary file 1 — Additional file 1: Additional methods: Radio tracking and weather data. Figure S1. Radio-telemetry system on Helgoland and example of signals received during the departure of a radio tracked northern wheatear. Figure S2. Variation in nocturnal departure timing as observed in Northern Wheatears, European Robins and Common Blackbirds during autumn. Table S1. Variation in explanatory variables used to explain night-to-night and within-night departure decisions in the three investigated migratory songbird species. Table S2. Comparison of candidate binary logistic regression models to assess the effect of fuel load on birds’ departure probability during the first night following capture (night-to-night departure decision). Table S3. Comparison of candidate time-dependent Cox proportional hazards models to assess the effects of weather variables on birds’ departure probability (night-to-night departure decision). Table S4. Comparison of candidate beta regression models to assess the effects of weather variables on birds’ nocturnal departure timing (proportion of night at departure; within-night departure decision). Strategy-specific differences in birds’ night-to-night and within-night departure decisions: Modelling approaches, Results. [file 40462_2020_193_MOESM1_ESM.pdf]

Additional file 1

Stopover departure decisions in songbirds:

Do long-distance migrants depart earlier and more independently of weather conditions than medium-distance migrants?

Florian Packmor[1,2], **Thomas Klinner**[1], **Bradley K. Woodworth**[3], **Cas Eikenaar**[1] & **Heiko Schmaljohann**[1,4]

[1] Institute of Avian Research "Vogelwarte Helgoland", An der Vogelwarte 21, 26386 Wilhelmshaven, Germany

[2] School of Natural Sciences, Bangor University, Deiniol Road, Bangor LL57 2UW, United Kingdom

[3] School of Biological Sciences, The University of Queensland, Brisbane 4072, Queensland, Australia

[4] Institute for Biology und Environmental Sciences, Carl von Ossietzky University of Oldenburg, Carl-von-Ossietzky-Straße, 26129 Oldenburg, Germany

Additional methods:

Radio tracking

In order to determine the timing of individual departures, birds were fitted with uniquely coded radio-transmitters (NTQB-1 Avian Nano Tag; weight: 0.29 g; burst interval: 2 – 4 sec; Lotek Wireless Inc., Newmarket, ON, Canada) using leg-loop harnesses adjusted to body size [1]. Mass of radio tags including harness(ca. 0.34 g) did not exceed 2 % (Wheatears; min. mass: 18.5 g), 2.6 % (Robins; min. mass: 14.4 g) or 0.5 % (Blackbirds; min. mass: 83.4 g) of the individual birds' body mass, respectively [2]. We established an automated digital radio-telemetry system ([3]; www.motus.org) that consists of four telemetry towers at three sites on Helgoland, each equipped with a SensorGnome receiver (www.sensorgnome.org) and three antennas (6EL Yagi antennas; Vårgårda Radio AB, Sweden; [4]). The overall array of 12 horizontally mounted antennas was aligned radially at intervals of approx. 30° [4]). The radio-telemetry system

continuously recorded radio signals on the utilized frequency (150.1 MHz) during the study periods to determine the timing of individual departure events.

Departures as obtained by the system are generally characterized by a rapid increase in signal strength detected from all/most antennas (bird is setting off the ground), followed by a decline in signal strength from a decreasing number of antennas until the loss of signal (bird is leaving the site towards a specific direction). We used the recorded data to determine time of take-off for each bird, defined as the time of highest signal strength during each departure event. Based on the time of take-off we calculated the respective temporal difference between initial capture and departure (minimum stopover duration in days), the binary departure decisions of the birds for each day/night they were present on Helgoland (staying vs. departing), as well as birds' nocturnal departure timing in relation to night length (proportion of night at departure). Departure directions of the individual radio-tagged birds were estimated by calculating a weighted circular mean of the directions the receiving antennas were aligned to. We excluded signals from the first half of the departure event to reduce the chance of taking misleading detections from antennas' back and side lobes into account. Directions of signals included in the circular mean were weighted by their temporal proximity to the last detection. Whenever pivotal antennas (antennas aligned to a direction close to the calculated departure direction) failed recording signals during the departure event and/or the signal got lost shortly after birds' take-off (<3 minutes) the obtained departure directions were discarded, as these were probably imprecise.

All tracking data were inspected visually. If the specific departure pattern described above was missing, we did not ascertain departure time. This was the case in 16 of the 97 Wheatears, 23 of the 54 Robins and 17 of the 71 Blackbirds radio-tagged for this study. Since we could not exclude that these birds were caught by a predator during stopover or that their radio-transmitters dropped or stopped transmitting (technical failure, battery life), they were omitted from all analyses.

Weather data

We used NCEP reanalysis data provided by the National Oceanic and Atmospheric Administration (NOAA; Boulder, CO, USA; http://www.cdc.noaa.gov/cdc/data.ncep.reanalysis.derived.html; [5]) to estimate the specific wind conditions the individual birds experienced during their stopover on Helgoland, and at the time of their individual departure. Data (U and V wind components) were obtained via the "RNCEP" R-package [6]) for a pressure level of 1000 mbar ("close to surface"). Speed and direction of the wind were interpolated with regard to the study site and both time of sunset for each day a bird stayed on Helgoland and time of individual departure [6]). We decided to use the NCEP reanalysis data instead of the wind data provided by the weather stations on Helgoland, as near-ground wind measurements taken by the

latter are likely biased by topography-induced turbulences. Tailwind assistance [m/s] towards the species-specific mean departure direction (Wheatears: 176°, rho = 0.73, $p < 0.001$, n = 66, range: 109° - 270°; Robins: 138°, rho = 0.48, $p < 0.001$, n = 30, range: 53° - 335°; Blackbirds: 181°, rho = 0.52, $p < 0.001$, n = 50, range: 70° - 300°) was calculated for each bird, both at time of sunset for each day it stayed on Helgoland and at time of its individual departure, using the $EQ^{Tailwind}$ [7] implemented in the function "NCEP.Airspeed()" [6] as follows:

$$Tailwind\ assistance_{\mathrm{i}} = windspeed_{\mathrm{i}} * \cos(wind\ direction_{\mathrm{i}} - mean\ departure\ direction)$$

We decided to use tailwind assistance instead of other, more sophisticated, measures of air flow assistance during flight (e.g. $EQ^{Airspeed}$; [7]), because some birds included in our study experienced crosswinds with a speed equal to the assumed species-specific air speed [8], which is incompatible with the underlying equation of the latter. Additionally, we calculated the crosswind [absolute values in m/s] perpendicular to the species-specific mean departure direction (see above) each bird experienced at time of sunset for each day it stayed on Helgoland and at time of its individual departure using the function "NCEP.Airspeed()" [6].

Other meteorological data were obtained from an automated weather station on Helgoland operated by the German Meteorological Office (DWD; ftp://ftp-cdc.dwd.de/pub/CDC/observations_germany/ climate/hourly/). We used these measurements to assign atmospheric pressure [mbar], air temperature [°C], and cloud cover [x/8] at both time of sunset for each day a bird stayed on Helgoland and individual nocturnal departure time. As these data include hourly measurements, we assigned the last measurement before either sunset and/or departure. During autumn 2017 the automated barometer of the weather station did not record atmospheric pressure for approximately one week, which coincided with the departures of ten Robins. In order to fill this gap, we downloaded site-specific atmospheric pressure estimates from the NOAA (http://www.cdc.noaa.gov/cdc/ data.ncep.reanalysis.derived.html; [5]). We run linear regression models to compare these estimates with atmospheric pressure measurements from the weather station. In general, the atmospheric pressure estimates were slightly lower than the actual measurements. Thus, we used the results of a regression model to adapt the estimates to the level of the measurements as follows:

$$\text{Atmospheric pressure: } measurement_{\mathrm{i}} = 1.002 * estimate_{\mathrm{i}} + 1.89$$

(Linear regression: n = 121, F = 86740, $R^2 = 0.998$, $p < 0.0001$)

Additionally, we calculated the change in atmospheric pressure and air temperature as the differences between the last measurements prior to either sunset or time of departure and the respective measurements 24 h before.

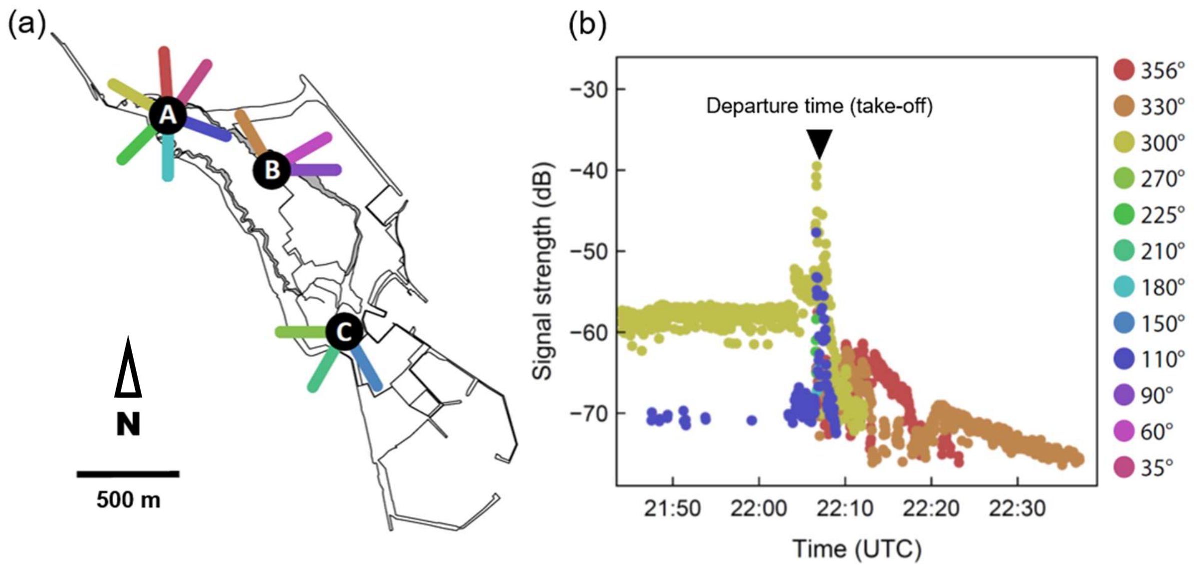

Figure S1. (a) Radio-telemetry system on Helgoland and (b) example of signals received during the departure of a radio tracked northern wheatear. (a) The radio-telemetry array consisting of 12 antennas at three sites (A, B, C). Coloured bars represent the different antennas and correspond to those given in (b). (b) Nocturnal departure event as recorded by the automated digital radio-telemetry system showing raw signal strength data against time (Coordinated Universal Time: UTC). The time of departure (take-off) defined as time of highest signal strength is given. Colours denote signals received by different antennas aligned to directions given in the key. Adapted from [4].

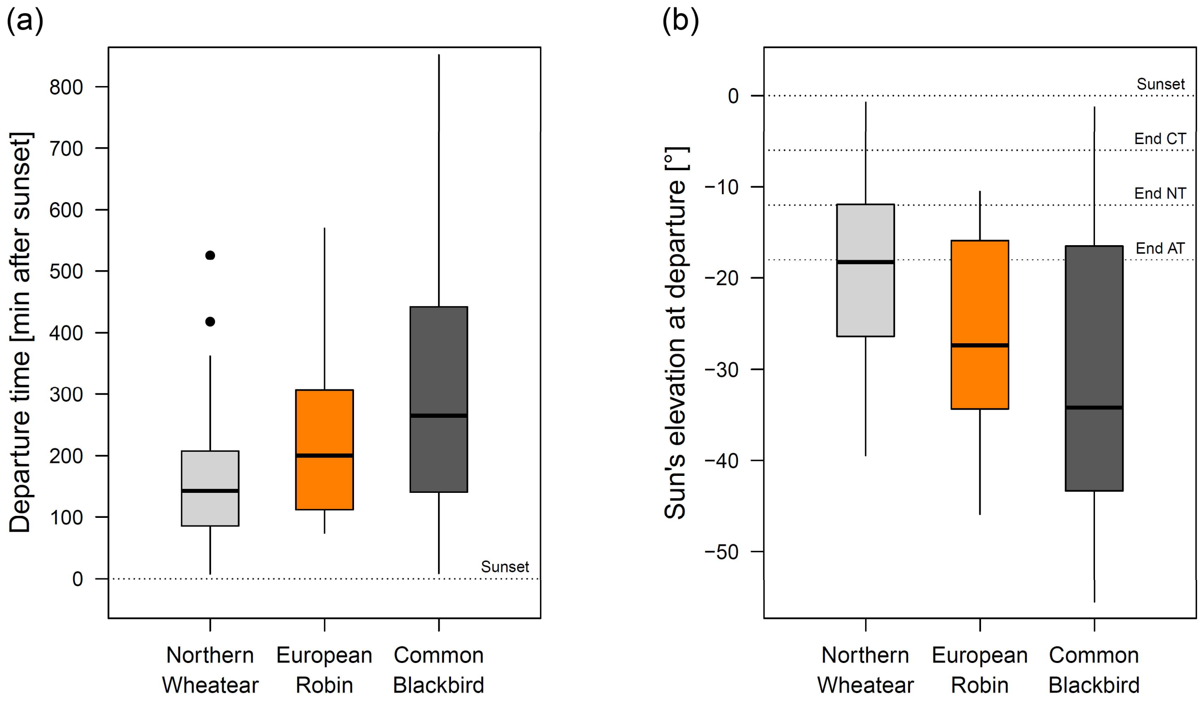

Figure S2. Variation in nocturnal departure timing as observed in Northern Wheatears, European Robins and Common Blackbirds during autumn. Nocturnal departure timing is expressed as (a) departure time [minutes after sunset] and (b) sun's elevation at departure [°]. Box plots show the 5th, 25th, 50th, 75th and 95th percentile as well as outliers (dots). Sample sizes are 75 (Northern Wheatear), 31 (European Robin) and 54 (Common Blackbird).

References:

1. Naef-Daenzer B. An allometric function to fit leg-loop harnesses to terrestrial birds. *J Avian Biol*. 2007; 38: 404-407.
2. Kenward RE. *A Manual for Wildlife Radio Tagging*. London, San Diego: Academic Press. 2001.
3. Taylor PD, Crewe TL, Mackenzie SA, Lepage D, Aubry Y, Crysler Z, Finney G, Francis CM, Guglielmo CG, Hamilton DJ, Holberton RL, Loring PH, Mitchell GW, Norris DR, Paquet J, Ronconi RA, Smetzer JR, Smith PA, Welch LJ, Woodworth BK. The Motus Wildlife Tracking System: a collaborative research network to enhance the understanding of wildlife movement. *Avian Conserv Ecol.* 2017; 12: 8.
4. Müller F, Eikenaar C, Crysler ZJ, Taylor PD, Schmaljohann H. Nocturnal departure timing in songbirds facing distinct migratory challenges. *J Anim Ecol*. 2018; 87: 1102-1115.
5. Kalnay E, Kanamitsu M, Kistler R, Collins W, Deaven D, Gandin L, Iredell M, Saha S, White G, Woollen J, Zhu Y, Leetmaa A, Reynolds R, Chelliah M, Ebisuzaki W, Higgins W, Janowiak J, Mo KC, Ropelewski C, Wang J, Jenne, Joseph D. The NCEP/NCAR 40-year reanalysis project. *Bull Am Meteorol Soc*. 1996; 77: 437-471.
6. Kemp MU, van Loon EE, Shamoun-Baranes J, Bouten W. RNCEP: global weather and climate data at your fingertips. *Methods Ecol Evol*. 2012; 3: 65-70.
7. Kemp MU, Shamoun-Baranes J, van Loon EE, McLaren JD, Dokter AM, Bouten W. Quantifying flow-assistance and implications for movement research. *J Theor Biol*. 2012; 308: 56-67.
8. Bruderer B, Boldt A. Flight characteristics of birds: I. radar measurements of speeds. *Ibis*. 2001; 143: 178-204.

Table S1. Variation in explanatory variables used to explain night-to-night and within-night departure decisions in the three investigated migratory songbird species (Northern Wheatear, European Robin and Common Blackbird). Given are the minimum (min.), 10th, 25th, 50th, 75th and 90th percentile as well as the maximum (max.) of the variation in each variable.

		Analyses of night-to-night departure decisions							Analyses of within-night departure decisions						
Species	Variable	Min.	10th percentile	25th percentile	Median	75th percentile	90th percentile	Max.	Min.	10th percentile	25th percentile	Median	75th percentile	90th percentile	Max.
Northern Wheatear	Fuel load	-0.13	-0.07	0.00	0.08	0.17	0.20	0.40	-0.13	-0.07	0.00	0.08	0.17	0.20	0.40
	Cloud cover [x/8]	0/8	0/8	2/8	7/8	8/8	8/8	8/8	0/8	0/8	0/8	3/8	8/8	8/8	8/8
	Tailwind assistance [m/s]	-7.4	-4.7	-2.8	-1.2	2.3	5.4	6.2	-6.0	-4.0	-1.9	-0.3	3.1	5.4	6.3
	Crosswind [absolute value in m/s]	0.2	0.8	1.2	3.3	6.5	9.3	11.6	0.0	0.7	1.2	2.7	6.1	7.7	10.1
	Atmospheric pressure [mbar]	1000	1010	1012	1019	1024	1035	139	1010	1013	1017	1021	1032	1039	1039
	Δ atmospheric pressure [mbar]	-11.7	-8.5	-3.0	0.3	6.3	7.5	15.6	-10.3	-4.8	-2.4	1.6	6.6	8.2	18.4
	Air temperature [°C]	9.2	13.7	14.7	16.0	18.4	20.1	22.4	9.5	14.2	14.4	14.8	18.4	19.8	21.5
	Δ air temperature [°C]	-3.1	-2.0	-1.2	-0.1	1.0	1.5	3.2	-3.6	-1.4	-0.7	-0.1	0.3	0.9	3.0
	Day of year [1 = 01 Jan.]	241	250	258	269	274	280	292	241	249	256	270	273	281	292
European Robin	Fuel load	-0.04	0.02	0.04	0.11	0.18	0.20	0.23	-0.04	0.02	0.04	0.11	0.18	0.20	0.23
	Cloud cover [x/8]	0/8	0/8	5/8	7/8	8/8	8/8	8/8	0/8	0/8	1/8	8/8	8/8	8/8	8/8
	Tailwind assistance [m/s]	-10.6	-6.8	-3.9	-1.1	0.7	2.9	8.8	-10.3	-3.4	-1.0	0.0	1.8	7.5	9.6
	Crosswind [absolute value in m/s]	0.1	0.4	0.8	3.3	6.0	8.7	12.3	0.1	0.9	2.2	3.3	4.6	7.5	9.5
	Atmospheric pressure [mbar]	990	998	1010	1016	1021	1024	1026	1006	1011	1015	1017	1024	1024	1025
	Δ atmospheric pressure [mbar]	-13.9	-5.7	-1.1	1.7	2.5	7.7	12.7	-10.8	-6.4	-2.4	1.7	3.1	10.2	13.2
	Air temperature [°C]	12.0	13.4	14.8	15.8	16.1	16.3	17.3	12.7	13.7	14.3	15.6	16.1	16.1	16.5
	Δ air temperature [°C]	-2.9	-1.0	-0.3	0.0	0.6	0.9	2.5	-1.7	-0.5	-0.4	0.0	0.4	0.9	2.5
	Day of year [1 = 01 Jan.]	249	255	260	266	272	282	292	259	263	266	273	279	283	292
Common Blackbird	Fuel load	-0.07	-0.01	0.05	0.12	0.21	0.25	0.40	-0.07	-0.01	0.05	0.12	0.21	0.26	0.40
	Cloud cover [x/8]	0/8	5/8	6/8	8/8	8/8	8/8	8/8	0/8	1/8	2/8	7/8	8/8	8/8	8/8
	Tailwind assistance [m/s]	-11.0	-5.3	-4.2	0.9	2.6	6.5	8.7	-4.2	-2.3	-0.2	2.1	5.9	7.5	7.7
	Crosswind [absolute value in m/s]	0.1	1.7	3.0	5.8	7.3	10.7	14.1	0.4	1.4	2.2	4.4	6.3	9.5	13.6
	Atmospheric pressure [mbar]	985	1007	1013	1015	1024	1027	1031	993	1010	1015	1018	1027	1030	1031
	Δ atmospheric pressure [mbar]	-17.0	-9.9	-3.0	-0.1	2.4	7.8	16.3	-11.3	-4.9	-1.5	2.4	7.8	9.2	10.1
	Air temperature [°C]	2.6	8.5	9.8	10.7	12.0	13.8	14.2	2.6	7.1	8.2	9.0	10.3	12.5	14.3
	Δ air temperature [°C]	-3.3	-2.5	-0.9	-0.4	1.0	2.0	6.9	-3.3	-2.8	-2.3	-1.2	-0.3	0.9	2.9
	Day of year [1 = 01 Jan.]	286	290	294	299	306	312	329	287	296	299	302	307	313	329

Table S2. Comparison of candidate binary logistic regression models to assess the effect of fuel load on birds' departure probability during the first night following capture (night-to-night departure decision). Models' coefficients and presence of factors are given. Degrees of freedom (df), second-order Akaike's information criterion values (AIC_c), AIC_c differences (Δ_i) and AIC_c weights (ω_i).

Model	Species	Fuel load	Day of year	df	AIC_c	Δ_i AIC_c	ω_i
1	+	0.866		4	175.6	0.00	0.43
2	+	0.918	-0.257	5	176.0	0.36	0.36

Table S3. Comparison of candidate time-dependent Cox proportional hazards models to assess the effects of weather variables on birds' departure probability (night-to-night departure decision). Models' coefficients and presence of factors are given. Degrees of freedom (df), second-order Akaike's information criterion values (AIC_c), AIC_c differences (Δ_i) and AIC_c weights (ω_i).

Model	Species	Cloud cover	Tailwind assistance	Cross-wind	Atmospheric pressure	Δ atmospheric pressure	Air temperature	Day of year	Species x tailw. assist.	Species x Δ atmosph. press.	Species x air temp.	df	AIC_c	Δ_i AIC_c	ω_i
1	+	-0.149	0.074	-0.321	0.158		0.339	0.324	+		+	12	1312.5	0.00	0.12
2	+	-0.176	0.132	-0.342			0.307	0.329	+		+	11	1312.6	0.14	0.11
3	+	-0.192	0.068	-0.322		0.167	0.337	0.348	+	+	+	14	1313.2	0.72	0.08
4	+		0.111	-0.319	0.192		0.387	0.311	+		+	11	1313.3	0.79	0.08
5	+	-0.174	0.099	-0.328		0.095	0.323	0.334	+		+	12	1313.8	1.26	0.06
6	+	-0.170	0.025	-0.303	0.146	0.142	0.364	0.343	+	+	+	15	1313.9	1.39	0.06

Table S4. Comparison of candidate beta regression models to assess the effects of weather variables on birds' nocturnal departure timing (proportion of night at departure; within-night departure decision). Models' coefficients and presence of factors are given. Degrees of freedom (df), second-order Akaike's information criterion values (AIC_c), AIC_c differences (Δ_i) and AIC_c weights (ω_i).

Model	Species	Cloud cover	Tailwind assistance	Cross-wind	Atmospheric pressure	Δ air temperature	Day of year	Species x cloud cover	Species x tailw. assist.	df	AIC_c	Δ_i AIC_c	ω_i
1	+	0.251	-0.185						+	8	-146.54	0.00	0.21
2	+	0.266	-0.251							6	-146.41	0.13	0.20
3	+	0.260	-0.232			0.057				7	-144.85	1.69	0.09
4	+	0.269	-0.253	-0.049						7	-144.76	1.74	0.09
5	+	0.229	-0.153		-0.052				+	9	-144.76	1.78	0.09
6	+	0.134	-0.160					+	+	10	-144.74	1.80	0.09
7	+	0.150	-0.233					+		8	-144.72	1.82	0.08
8	+	0.269	-0.237				-0.042			7	-144.61	1.93	0.08
9	+	0.255	-0.199	-0.034					+	9	-144.55	1.99	0.08

Strategy-specific differences in birds' night-to-night and nocturnal departure decisions

Modelling approaches:

We followed parallel modelling approaches focussing on either species- or strategy-specific differences in the birds' night-to-night and nocturnal departure decisions departure decisions, with Wheatears representing the long-distance migration strategy, and Robins and Blackbirds together representing the medium-distance migration strategy in the latter. Initial models fitted for assessing species- and strategy-specific differences were generally the same, only differing in the inclusion of either species or strategy as explanatory variable. Models including species are described in the method section of the main text, models including strategy are described below. We conducted an automated model selection for modelling approaches with two or more explanatory variables included in the initial model, which is described in the method section of the main text. We provide average estimates and corresponding 95 % confidence intervals for all explanatory variables included in the selected models with a ΔAICc <2.

Night-to-night departure decisions:

We analysed whether the minimum stopover duration of the medium-distance migrants differed from those of the long-distance migrants using a Poisson regression model (generalised linear model) with strategy (categorical: two levels: long-distance migrant and medium-distance migrant) as explanatory variable.

The effects of fuel load and weather variables on departure probability were analysed using two different modelling approaches. This was necessary, because all fuel load estimates were based on the birds' body masses at capture and get less reliable with each day they spent at the study site. The modelling approach

involving data on fuel load was, therefore, restricted to the departure probability during the first night following capture. Both modelling approaches are detailed below:

1. We assessed the effect of fuel load on departure probability during the first night following capture by fitting binary logistic regression models. The initial model included fuel load (continuous), strategy, day of year (1 January = 1; continuous), and the two-way interaction between fuel load and strategy as explanatory variables. Variables included in the selected models are detailed in Table S9.
2. We assessed the effect of weather variables on night-to-night departure probability using time-dependent Cox proportional hazards models, which describe the probability of an event (here 'departure') occurring over time as a function of a baseline probability (hazard) and a set of fixed or time-varying explanatory variables. We estimated the departure probability as a function of strategy (fixed variable), day of year (time-varying variable), and a set of weather variables (time-varying variables). Weather variables included in the initial model were tailwind assistance (continuous), crosswind (continuous), cloud cover (proportional), atmospheric pressure (continuous), change in atmospheric pressure (Δ atmospheric pressure; continuous), air temperature (continuous), and change in air temperature (Δ air temperature; continuous). Additionally, the initial model included the two-way interactions between strategy and each of the different weather variables. Variables included in the selected models are detailed in Table S10.

Nocturnal departure decisions:

The effects of fuel load and weather variables on birds' nocturnal departure timing were analysed in two different modelling approaches. The modelling approach involving data on fuel load was restricted to the nocturnal departure timing during the first night following capture for the same reason as described above. Both modelling approaches are detailed below:

1. We assessed the effect of fuel load on nocturnal departure timing (proportion of night at departure) of birds that left Helgoland during the first night following capture by fitting beta regression models using the “betareg” function implemented in the “betareg” package. The initial model included fuel load, strategy, day of year, and the two-way interaction between fuel load and strategy as explanatory variables. Variables included in the selected models are detailed in Table S11.
2. We assessed the effects of weather variables on nocturnal departure timing (proportion of night at departure) by fitting beta regression models, which included birds leaving Helgoland during the first or any other night following capture. The initial model included strategy, day of year, tailwind assistance, crosswind, cloud cover, atmospheric pressure, Δ atmospheric pressure, air temperature, Δ air temperature, and the two-way interactions between strategy and each of the different weather variables. Variables included in the selected models are detailed in Table S12.

Strategy-specific differences in birds' night-to-night and nocturnal departure decisions

Results:

The long- and medium-distance migrants differed significantly in their minimum stopover duration on Helgoland during autumn (Poisson regression model: Intercept: 0.75 (SE 0.08), $P < 0.001$; Strategy (medium-distance migrant): 0.99 (SE 0.09), $P < 0.001$; n = 160).

The results from all remaining modelling approaches are given in the Tables S5 – S8 (see below).

Table S5. Effects of fuel load and day of year on departure probability during the first night following capture in long-distance migrants (Northern Wheatears) and medium-distance migrants (European Robins and Common Blackbirds). Average model estimates, adjusted standard errors (SE), 95 % confidence intervals (CIs) and associated *p*-values of parameters included in the candidate models in Table S9 are shown. *P*-values < 0.05 are given in bold font. Reference category for species is Northern Wheatear.

**Parameter**	**Estimate±SE**	**95% CI**	***p***
**Intercept**	0.410±0.261	-0.106/0.925	0.120
**Strategy (medium-distance migrant)**	-1.816±0.393	-2.592/-1.041	**<0.001**
**Fuel load**	1.013±0.308	0.405/1.620	**0.001**
**Day of year**	-0.283±0.198	-0.675/0.108	0.156
**Strategy (medium-distance migrant) x fuel load**	-0.573±0.429	-1.420/0.275	0.185

Table S6. Effects of weather variables and day of year on night-to-night departure probability in long-distance migrants (Northern Wheatears) and medium-distance migrants (European Robins and Common Blackbirds). Average model estimates, adjusted standard errors (SE), 95 % confidence intervals (CIs) and associated *p*-values of parameters included in the candidate models in Table S10 are shown. *P*-values < 0.05 are given in bold font. Reference category for species is Northern Wheatear.

Parameter	Estimate±SE	95% CI	*p*
**Strategy (medium-distance migrant)**	-1.353±0.195	-1.736/-0.971	**<0.001**
**Cloud cover**	-0.167±0.078	-0.321/-0.013	**0.033**
**Tailwind assistance**	0.088±0.135	-0.176/0.353	0.513
**Crosswind**	-0.338±0.102	-0.539/-0.138	**<0.001**
**Atmospheric pressure**	0.171±0.098	-0.021/0.362	**0.081**
**Δ atmospheric pressure**	0.070±0.084	-0.096/0.235	0.410
**Air temperature**	0.373±0.172	0.177/0.828	**0.030**
**Day of year**	0.339±0.109	0.125/0.533	**0.002**
**Strategy (medium-distance migrant) x tailwind assistance**	0.503±0.177	0.024/0.698	**0.004**
**Strategy (medium-distance migrant) x air temperature**	-0.491±0.209	-1.134/-0.356	**0.019**

Table S7. Effect of fuel load on nocturnal departure timing (proportion of night at departure) during the first night following capture in long-distance migrants (Northern Wheatears) and medium-distance migrants (European Robins and Common Blackbirds). Estimates, standard errors (SE), 95 % confidence intervals (CIs) and associated *p*-values of all parameters included in the candidate models in Table S11 are shown. *P*-values < 0.05 are given in bold font. Reference category for species is Northern Wheatear.

Parameter	Estimate±SE	95% CI	*p*
**Intercept**	-1.292±0.126	-1.539/-1.045	**<0.001**
**Strategy (medium-distance migrant)**	0.884±0.206	0.479/1.288	**<0.001**
**Fuel load**	-0.255±0.122	-0.493/-0.017	**<0.036**
**Strategy (medium-distance migrant) x fuel load**	-0.254±0.234	-0.717/0.206	0.279

Table S8. Effects of weather variables and day of year on nocturnal departure timing (proportion of night at departure) in long-distance migrants (Northern Wheatears) and medium-distance migrants (European Robins and Common Blackbirds). Average model estimates, adjusted standard errors (SE), 95 % confidence intervals (CIs) and associated *p*-values of parameters included in the candidate models in Table S12 are shown. *P*-values < 0.05 are given in bold font. Reference category for species is Northern Wheatear.

**Parameter**	**Estimate±SE**	**95% CI**	***p***
**Intercept**	-1.170±0.096	-1.359/-0.982	**<0.001**
**Strategy (medium-distance migrant)**	0.366±0.126	0.119/0.613	**0.004**
**Cloud cover**	0.178±0.105	0.099/0.657	0.090
**Tailwind assistance**	-0.189±0.081	-0.371/-0.065	**0.019**
**Crosswind**	0.057±0.105	-0.150/0.263	0.591
**Δ air temperature**	-0.013±0.063	-0.238/0.163	0.835
**Day of year**	-0.064±0.068	-0.197/0.070	0.348
**Strategy (medium-distance migrant) x cloud cover**	0.249±0.135	-0.015/0.513	0.064
**Strategy (medium-distance migrant) x tailwind assistance**	-0.123±0133	-0.383/0.137	0.354
**Strategy (medium-distance migrant) x crosswind**	-0.285±0140	-0.558/-0.011	**0.041**
**Strategy (medium-distance migrant) x Δ air temperature**	0.260±0.129	0.006/0.514	**0.045**

Table S9. Comparison of candidate binary logistic regression models to assess the effect of fuel load on the departure probability during the first night following capture (night-to-night departure decision) in long- and medium-distance migrants. Models' coefficients and presence of factors are given. Degrees of freedom (df), second-order Akaike's information criterion values (AIC_c), AIC_c differences (Δ_i) and AIC_c weights (ω_i).

Model	Strategy	Fuel load	Day of year	Strategy x fuel load	df	AIC_c	Δ_i AIC_c	ω_i
1	+	0.849			3	178.51	0	0.28
2	+	0.906	-0.260		4	178.74	0.24	0.25
3	+	1.227	-0.307	+	5	178.77	0.26	0.25
4	+	1.106		+	4	179.08	0.57	21

Table S10. Comparison of candidate time-dependent Cox proportional hazards models to assess the effects of weather variables on departure probability (night-to-night departure decision) in long- and medium-distance migrants. Models' coefficients and presence of factors are given. Degrees of freedom (df), second-order Akaike's information criterion values (AIC_c), AIC_c differences (Δ_i) and AIC_c weights (ω_i).

Model	Strategy	Cloud cover	Tailwind assistance	Cross-wind	Atmospheric pressure	Δ atmospheric pressure	Air temperature	Day of year	Strategy x tailw. assist.	Strategy x air temp.	df	AIC_c	Δ_i AIC_c	ω_i
1	+	-0.149	0.082	-0.321	0.165		0.375	0.355	+	+	9	1313.5	0.00	0.13
2	+	-0.174	0.143	-0.341			0.337	0.354	+	+	8	1314.0	0.48	0.10
3	+		0.121	-0.322	0.194		0.421	0.341	+	+	8	1314.4	0.88	0.08
4	+	-0.196	-0.020	-0.409				0.267	+		6	1314.5	0.97	0.08
5	+	-0.169	0.117	-0.320		0.088	0.357	0.358	+	+	9	1315.1	1.60	0.06
6	+	-0.150	0.076	-0.311	0.146	0.047	0.381	0.357	+	+	10	1315.5	1.99	0.05

Table S11. Comparison of candidate beta regression models to assess the effect of fuel load on nocturnal departure timing (proportion of night at departure; within-night departure decision) in long- and medium-distance migrants. Models' coefficients and presence of factors are given. Degrees of freedom (df), second-order Akaike's information criterion values (AIC_c), AIC_c differences (Δ_i) and AIC_c weights (ω_i).

Model	Strategy	Fuel load	Strategy x fuel load	df	AIC_c	Δ_i AIC_c	ω_i
1	+	-0.284		4	-74.55	0	0.65
2	+	-0.203	+	5	-73.35	1.19	0.35

Table S12. Comparison of candidate beta regression models to assess the effects of weather variables on nocturnal departure timing (proportion of night at departure; within-night departure decision) in long- and medium-distance migrants. Models' coefficients and presence of factors are given. Degrees of freedom (df), second-order Akaike's information criterion values (AIC_c), AIC_c differences (Δ_i) and AIC_c weights (ω_i).

Model	Strategy	Cloud cover	Tailwind assistance	Cross-wind	Δ air temperature	Day of year	Strategy x cloud cover	Strategy x tailw. assist.	Strategy x crosswind	Strategy x Δ air temp.	df	AIC_c	Δ_i AIC_c	ω_i
1	+	0.157	-0.198	0.054			+		+		8	-149.7	0	0.14
2	+	0.148	-0.163	0.068	-0.061		+		+	+	10	-149.6	0.11	0.13
3	+	0.150	-0.233				+				6	-149.1	0.65	0.1
4	+	0.270	-0.173	0.095	-0.053				+	+	9	-148.7	0.98	0.09
5	+	0.266	-0.250								5	-148.5	1.19	0.08
6	+	0.129	-0.233	-0.084			+				7	-148.5	1.26	0.08
7	+	0.167	-0.171	0.076		-0.065	+		+		9	-148.4	1.37	0.07
8	+	0.162	-0.179	0.058	0.062		+		+		9	-148.3	1.47	0.07
9	+	0.149	-0.135	0.075			+	+	+		9	-148.2	1.56	0.07
10	+	0.156	-0.139	0.088	-0.073	-0.063	+		+	+	11	-148.1	1.6	0.06
11	+	0.133	-0.159				+	+			7	-147.9	1.78	0.06
12	+	0.301	-0.221	0.082					+		7	-147.8	1.89	0.05
